# Anthropometric and clinical correlates of fat mass in healthy term infants at 6 months of age

**DOI:** 10.1186/s12887-019-1430-x

**Published:** 2019-02-18

**Authors:** Ameyalli M. Rodríguez-Cano, Jennifer Mier-Cabrera, Cinthya Muñoz-Manrique, Arturo Cardona-Pérez, Gicela Villalobos-Alcázar, Otilia Perichart-Perera

**Affiliations:** 1 Nutrition and Bioprogramming Department, Instituto Nacional de Perinatología “Isidro Espinosa de los Reyes”, Montes Urales 800, Col. Lomas de Virreyes, 11000 Ciudad de México, CP Mexico; 2 Neonatal Ward, Instituto Nacional de Perinatología “Isidro Espinosa de los Reyes”, Montes Urales 800, Col. Lomas de Virreyes, 11000 Ciudad de México, CP Mexico; 3 General Director, Instituto Nacional de Perinatología “Isidro Espinosa de los Reyes”, Montes Urales 800, Col. Lomas de Virreyes, 11000 Ciudad de México, CP Mexico

**Keywords:** Body composition, Air displacement plethysmography, Fat Mass Index, Skinfolds, Adiposity, Hispanic, Type of feeding

## Abstract

**Background:**

Body composition in infancy plays a central role in the programming of metabolic diseases. Fat mass (FM) is determined by personal and environmental factors. Anthropometric measurements allow for estimations of FM in many age groups; however, correlations of these measurements with FM in early stages of life are scarcely reported. The aim of this study was to evaluate anthropometric and clinical correlates of FM in healthy term infants at 6 months of age.

**Methods:**

Healthy term newborns (*n* = 102) from a prospective cohort. Weight, length, skinfolds (biceps, triceps, subscapular and the sum -SFS-) and waist circumference (WC) were measured at 6 months. Body mass index (BMI) and WC/length ratio were computed. Type of feeding during the first 6 months of age was recorded. Air displacement plethysmography was used to asses FM (percentage -%-) and FM index (FMI) was calculated. Correlations and general linear models were performed to evaluate associations.

**Results:**

Significant correlations were observed between all anthropometric measurements and FM (% and index)(*p* < 0.001). Exclusive/predominant breastfed infants had higher FM and anthropometric measurements at 6 months. Models that showed the strongest associations with FM (% and index) were SFS + WC + sex + type of feeding.

**Conclusions:**

Anthropometry showed good correlations with FM at 6 months of age. Skinfolds sum and waist circumference were the strongest anthropometric variables associated to FM. Exclusive/predominant breastfeeding was strongly associated with FM.

## Background

Worldwide increasing prevalence of overweight and obesity among children has highlighted the need for better strategies in its early detection and prevention. The 2013 Global Burden of Disease Study showed a rise of 47% in overweight and obesity prevalence in children since 1980 [1]. Maternal factors, such as pre-gestational obesity and gestational weight gain, have been identified to influence the risk of obesity and the development of fat mass (FM) in the offspring [2]. Postnatal environmental factors, such as type of feeding (breastfeeding vs formula) and duration of breastfeeding, have been recognized as determinants of body composition in infants [3, 4]. Some authors have reported that exclusively breastfed infants appear to have higher body fat when compared to formula fed ones during the first 6 months of age [5–7].

Because body composition in early infancy could play a central role in the programming of metabolic diseases [8, 9], accurate measurement of FM should be part of an infant’s nutrition assessment since birth and throughout life [10–12]. Air displacement plethysmography (ADP) is a valid method for assessing FM in infants [13, 14]. However, since ADP is not widely accessible and is often not practical, the estimation of body composition is generally performed through surrogate methods. Anthropometric measurements have been widely used in other age groups because it is a relatively easy, simple and inexpensive alternative for estimating adiposity in clinical settings [11, 15]. There are few data about the association of anthropometry and FM estimation in the first 6 months of age [16–18].

Weight-for-length (W/L) and body-mass-index for age (BMI/A) are anthropometric indices used for the diagnosis of overweight and obesity in children [19]. The evidence shows that these are valid indicators to estimate body fat in school-age children and adolescents [20–22], but it remains unclear about their use in infants.

Therefore, the aim of this study was to evaluate how anthropometric measurements and clinical factors correlate with FM at 6 months of age in healthy term Mexican infants.

## Methods

### Study settings

This descriptive study derives from a prospective cohort of pregnant women conducted at the National Institute of Perinatology (Mexico City, 2009–2012).

### Subjects’ recruitment

We consecutively selected women in their first trimester of pregnancy (< 14 weeks), who did not have comorbidities (diabetes mellitus, renal, hepatic and/or autoimmune diseases), did not use medication that affected metabolism (metformin, prednisone, etc.) and did not have multiple pregnancies. All adult women that accepted to participate signed an informed consent; for those < 19 years old, the consent of parents was also required. Women were followed monthly until the end of pregnancy.

### Participants’ selection

Newborns were selected by convenience from healthy mothers participating in the afore-mentioned cohort. We invited to participate only those mother-baby binomials that did not develop gestational diabetes or preeclampsia and whose babies were born at the institutional hospital. We included healthy term newborns (≥37 weeks). Those babies born prematurely (< 37 weeks) or with congenital/metabolic diseases were excluded. Babies that lacked body composition assessment at 6 months were eliminated from the analysis.

### Anthropometry

A nutritionist with prior experience and adequately trained in anthropometric measurement standardization performed all the babies’ anthropometric procedures at birth (first 72 h) and at 6 months of age, following the methods described by Lohman [23].

Infants were measured without clothes. The average of two measurements was recorded, except for weight and FM. At birth, weight was recorded in grams to the nearest 0.1 kg using a pediatric scale (1582 Baby/Mommy Scale, TANITA Corp., Tokyo, Japan). Low birth weight (< 2500 g) and macrosomia (> 4000 g) were recorded. At 6 months, weight was measured using the digital scale from the PEAPOD Infant Body Composition System (software version 3.1.0, Lifestyle Measurement Instruments, COSMED, California, USA). Recumbent length was measured to the nearest 0.1 cm using an infantometer (SECA 207, SECA, Hamburg, Germany). With the help of a nurse, the infant was laid down and her/his head was placed in the Frankfort plane (as the best anatomical indicator of a physiological position for a population without facial deformities) [24]. Length was measured from the crown of the head to the foot’s heel, while both were appropriately attached to the infantometer. BMI was computed (weight/length^2^) and sex-specific z-scores for BMI/A and W/L were calculated using the World Health Organization (WHO) Anthro software program (version 3.0.1, WHO, Geneva, Switzerland). Risk of overweight (> 1 standard deviation scores), overweight (> 2 standard deviation scores) and obesity (> 3 standard deviation scores) was defined based on z-scores.

Waist circumference (WC) was measured around the umbilicus to the nearest 0.1 cm with a fiberglass anthropometric measuring tape (Gulick, Sammons Preston, Chicago, USA). WC-to-length ratio (WLR) was obtained dividing WC by length.

Skinfolds (biceps –BSF–, triceps –TSF– and subscapular –SSF) were measured with a Lange caliper (Beta Technology, California, USA) from the child’s left arm holding the skin between the index finger and thumb (one centimeter above the measurement site) while the baby was laid down. BSF and TSF were measured taking as reference the mid-point of the arm grasping the skin parallel to the floor. SSF was taken at a 45° to the horizontal plane. A difference > 2 mm between both measurements required a third measurement. Skinfold sum (SFS) was obtained by adding the values from the three skinfolds aforementioned.

### Fat mass (FM% and FMI)

The assessment of FM (percentage -%- and kilograms -kg-) and fat-free mass (FFM, % and kg) was performed at 6 months using the PEAPOD Infant Body Composition System [25, 26]. Every day, before starting measurements, the PEAPOD was calibrated according to the manufacturer’s protocol. Before placing the infant in the PEAPOD scale, (s) he was undressed and a head cap was put on to minimize air trapped in the hair. Then, (s) he was placed in the test chamber tray in order to start with the measurement of body composition. After volume and weight were measured by de PEAPOD, the system’s software calculated the infant’s body composition based on Fomon’s body density values. FMI (FMkg/length^2^) was computed to correct for variations in FM related to body size [27].

### Feeding practices assessment

At 1-, 3- and 6-months-old visits, mothers were asked a series of questions regarding their infant’s feeding practices. Using the information collected from the 6-months questionnaire, infants were classified within 3 breastfeeding categories according to the WHO definition [28]: a) exclusive and predominant breastfed for 6 months (E/P BF), b) breastfed for up to 6 months + formula feeding (BF + F), and c) breastfed for less than 6 months + formula feeding (BF < 6 m + F).

### Maternal information

Mothers participating in the cohort study reported their age and pre-gestational weight in their first visit. Pre-gestational BMI was computed and classified according to WHO criteria (low, normal, overweight or obesity).

### Statistical analysis

Descriptive statistics and frequencies were performed for all variables. Mean differences were analyzed using Student’s t-test for parametric variables or U Mann-Whitney for non-parametric ones. One-way ANCOVA was performed to evaluate differences in anthropometric indices and FM according to type of feeding. Pearson’s and Spearman’s correlations were used to evaluate bivariate associations between FM (% and index) and anthropometric measures.

General linear models were performed to evaluate the association of anthropometric measurements (TSF, BSF, SSF, SFS, WC and length) and clinical factors with FM (% and index) at 6 months. The dependent variable was FM% or FMI; independent variables included sex and type of feeding. For FM% models, length was considered as a variable, and excluded in FMI models. A *p*-value < 0.05 was considered statistically significant. Statistics were performed with the IBM SPSS Software (version 22, SPSS Statistics/IBM Corp, New York, USA).

## Results

### Participants’ characteristics

A total of 222 healthy mother-baby binomials from the cohort study were recruited following birth and invited to participate. Nevertheless, only 155 agreed to participate. Fifty-three infants that lacked body composition measurement at 6 months were eliminated. This analysis included a total of 102 infants. Due to different circumstances not attributable to our staff, it was not possible to assess weight and length of eight infants at birth (*n* = 94) (Table 1).

**Table 1:** Descriptive data of mothers, newborns and infants at 6 months

Maternal information	ALL (*n* = 102)	GIRLS (*n* = 53)	BOYS (*n* = 49)
Age (years)	28.5 (14–44)	23 (14–40)^#^	32 (14–44)^#^
Pre-gestational weight (kg)	59 (50.4–68)	58.0 (50.0–68.8)	60.0 (53.5–67.5)
Height (cm)	156.7 ± 5.8	156.7 ± 6.6	156.8 ± 4.9
Pre-gestational BMI (kg/m^2^)	23.9 (15.1–48.4)	23.3 (15.1–35.3)	24.6 (18.4–48.1)
Overweight/obesity	41 (40.2)	19 (35.8)	22 (44.9)
GWG (3rd trimester)	*n* = 92	*n* = 49	*n* = 43
Adequate	22 (23.9)	10 (20.4)	12 (27.9)
Excessive	43 (46.7)	23 (46.9)	20 (46.5)
Infant anthropometry @ birth	*n* = 94	*n* = 49	*n* = 45
Weight (kg)	2.89 ± 0.35	2.81 ± 0.32*	2.98 ± 0.35*
Length (cm)	46.9 ± 2.0	46.4 ± 1.9*	47.5 ± 1.9*
BMI (kg/m^2^)	13.1 ± 1.0	13.1 ± 1.0	13.0 ± 1.1
BMI/A (z-score)	− 0.20 ± 0.84	− 0.16 ± 0.79	− 0.26 ± 0.90
W/L (z-score)	0.31 ± 0.95	0.39 ± 0.87	0.26 ± 1.03
Infant anthropometry @ 6 months	*n* = 102	*n* = 53	*n* = 49
Weight (kg)	7.13 ± 0.86	6.91 ± 0.81*	7.47 ± 0.86*
Length (cm)	64.0 ± 2.6	63.1 ± 2.2	65.0 ± 2.6
BMI (kg/m^2^)	17.4 ± 1.6	17.3 ± 1.6	17.4 ± 1.6
BMI/A (z-score)	0.10 ± 1.1	0.22 ± 1.00	− 0.02 ± 1.18
W/L (z-score)	0.26 ± 1.1	0.40 ± 1.00	0.12 ± 1.18
Waist circumference (cm)	39.4 ± 2.7	39.2 ± 2.7	39.7 ± 2.8
Triceps skinfold (mm)	8.0 (7.0–9.5)	8.0 (7.0–9.0)	8.0 (7.0–10.0)
Biceps skinfold (mm)	6.0 (5.0–7.0)	6.0 (4.0–7.0)	6.0 (5.0–7.0)
Subscapular skinfold (mm)	7.0 (6.0–9.0)	7.0 (6.0–9.0)	7.0 (6.5–8.0)
Skinfolds sum (mm)	21 (18.0–24.5)	21 (18.0–23.5)	21 (18.5–25.0)
Fat mass (%)	25.8 ± 5.9	26.0 ± 6.4	25.5 ± 5.5
Fat mass (kg)	1.9 ± 0.6	1.8 ± 0.6	1.9 ± 0.5
FMI (FMkg/length^2^)	4.5 ± 1.4	4.5 ± 1.5	4.5 ± 1.2
Fat-free mass (%)	74.2 ± 5.9	74.0 ± 6.4	74.5 ± 5.5
Fat-free mass (kg)	5.3 ± 0.6	5.1 ± 0.5**	5.5 ± 0.5**
Feeding information @ 6 months	*n* = 102	*n* = 53	*n* = 49
Exclusive/predominant breastfeeding	26 (25.5)	13 (24.5)	13 (26.5)
Breastfeeding up to 6 months + formula	47 (46.1)	22 (41.5)	25 (51.0)
Breastfeeding < 6 months + formula	29 (28.4)	18 (34.0)	11 (22.4)
Started complementary feeding < 4 month	25 (24.8)	15 (28.3)	10 (20.8)
Started complementary feeding > 4 month	76 (75.2)	38 (71.7)	38 (79.2)

Data are shown as Mean ± SD; Median (25°-75°); *n* (%)

*BMI* Body mass index, *GWG*, Gestational weight gain, *BMI/A* Body-mass-index/age, *W/L*, weight-to-length, *FMI* Fat mass index, *FMkg* kilograms of fat mass

T-Student test *p < 0.05; **p = 0.001 (girls vs boys); ^#^U Mann-Whitney test *p* < 0.05 (girls vs boys)

Mothers had a mean age of 27.1 ± 8.7 years and their mean pre-gestational BMI was 24.7 ± 5.2 kg/m^2^. The prevalence of pre-gestational overweight/obesity was 40.2%. At birth, infants’ mean gestational age, weight and length was 39.0 ± 1.1 weeks, 2.89 ± 0.35 kg and 46.9 ± 2.0 cm, respectively. Low birth weight was present in 10.6% (*n* = 10) of newborns and no infants were classified as macrosomic. Most of the newborns had normal BMI/A at birth (93.4%, *n* = 95), and there were no infants classified with obesity during the first 6 months of age.

Descriptive data of mothers, newborns and infants at 6 months is presented in Table 1. Boys had a higher weight and length at birth, as well as a higher weight and FFM at 6 months (*p* < 0.05). No other anthropometric differences were observed by sex (*p* > 0.05).

### Type of feeding, anthropometry and FM

FM (% and kg) was higher and FFM (% and kg) was lower in infants classified as E/P BF when compared to BF + F or BF < 6 m + F. Except for WC, all anthropometric measurements were higher in the E/P BF group (adjusted by sex, BMI/A at birth and pre-gestational BMI) (Table 2).

**Table 2:** Fat mass and anthropometric data according to type of feeding in infants at 6 months

	All *n* = 102	Exclusive/predominant Breastfeeding *n* = 26	Breastfeeding up to 6 months + Formula *n* = 47	Breastfeeding < 6 months + Formula *n* = 29
BMI (kg/m^2^)	17.4 ± 1.6	17.8 ± 2.0	17.3 ± 1.3	17.1 ± 1.6^a^
Waist circumference (cm)	39.4 ± 2.7	40.0 ± 3.1	39.3 ± 2.8	39.1 ± 2.2
Triceps skinfold (mm)	8.1 ± 1.9	8.6 ± 2.2	7.7 ± 1.6^a^	8.2 ± 1.9
Skinfolds sum (mm)	21.6 ± 4.5	23.2 ± 5.7	20.9 ± 3.6^a^	21.3 ± 4.4^a^
Fat mass (%)	25.8 ± 5.9	29.4 ± 5.6	24.9 ± 5.8^b^	23.9 ± 5.2^c^
Fat mass (kg)	1.9 ± 0.6	2.1 ± 0.6	1.8 ± 0.6^b^	1.7 ± 0.4^a^
FMI (FMkg/length^2^)	4.5 ± 1.4	5.3 ± 1.5	4.3 ± 1.3^b^	4.2 ± 1.1^c^

Data are shown as Mean ± SD

One-way ANCOVA. LSD post hoc test. ^a^*p* < 0.05, ^b^*p* < 0.01 and ^c^*p* < 0.001 vs Exclusive/predominant breastfeeding

Model adjusted by sex, BMI/A (z-score) at birth and pre-gestational BMI (kg/m^2^)

*BMI* Body mass index, *FMI* Fat mass index, *FMkg* kilograms of fat mass

### FM and anthropometric associations

Correlations of anthropometric measurements with FM% at 6 months were all statistically significant (*p* < 0.001). The strongest correlation coefficients were for BMI (*r* = 0.691), SFS (*r* = 0.603) and WC (r = 0.591), followed by WLR (*r* = 0.589), SSF (r = 0.588) and TSF (*r* = 0.581). The weakest correlations were for weight (*r* = 0.569) and BSF (r = 0.409).

All anthropometric measurements had significant correlations with FMI (*p* < 0.0001). In order of strength, correlation coefficients were as follows: BMI (*r* = 0.816), WLR (*r* = 0.693), WC (r = 0.691), weight (*r* = 0.668), SFS (*r* = 0.640), SSF (r = 0.631), TSF (r = 0.624) and BSF (*r* = 0.415).

The strongest models of FM% included SFS + WC + length (model 2) followed by TSF + WC + length (Model 4) (Table 3). Regarding FMI, the strongest associations were shown for SFS + WC (model 2) followed by TSF + WC (Model 4) (Table 4).

**Table 3:** Correlates of FM% in infants at 6 months

Variable	B	IC 95%	Eta partial squared	*p*	R^2^ (R^2^ adjusted)
Model 1					0.500 (0.463)
Skinfolds sum	0.823	0.611 – 1.035	0.387	< 0.0001	
Length	0.209	-0.164 – 0.582	0.013	0.268	
Sex [female]	3.844	0.415 – 7.272	0.050	0.028	
Exclusive/Predominant Breastfeeding	6.399	2.809 – 9.988	0.118	0.001	
Breastfeeding up to 6 months + Formula	3.740	0.572 – 6.907	0.055	0.021	
Breastfeeding < 6 months + Formula	Reference				
Model 2					0.552 (0.513)
Skinfold sum	0.572	0.319 – 0.825	0.178	< 0.0001	
Waist circumference	0.720	0.283 – 1.157	0.103	0.002	
Length	-0.090	-0.489 – 0.309	0.002	0.654	
Sex [female]	3.203	−0.085 – 6.492	0.039	0.056	
Exclusive/Predominant Breastfeeding	5.960	2.531 – 9.388	0.114	0.001	
Breastfeeding up to 6 months + Formula	2.964	-0.088 – 6.107	0.038	0.057	
Breastfeeding < 6 months + Formula	Reference				
Model 3					0.443 (0.401)
Triceps Skinfold	1.746	1.220 – 2.271	0.316	< 0.0001	
Length	0.301	-0.090 – 0.692	0.024	0.130	
Sex [female]	3.862	0.223 – 7.500	0.045	0.038	
Exclusive/Predominant Breastfeeding	6.292	2.498 – 10.087	0.103	0.001	
Breastfeeding up to 6 months + Formula	4.164	0.784 – 7.543	0.060	0.016	
Breastfeeding < 6 months + Formula	Reference				
Model 4					0.531 (0.491)
Triceps Skinfold	1.117	0.547 – 1.686	0.140	< 0.0001	
Waist circumference	0.883	0.464 – 1.303	0.158	< 0.0001	
Length	-0.102	-0.510 – 0.307	0.003	0.622	
Sex [female]	3.105	-0.271 – 6.842	0.035	0.071	
Exclusive/Predominant Breastfeeding	5.820	2.312 – 9.328	0.105	0.001	
Breastfeeding up to 6 months + Formula	3.086	-0.074 – 6.246	0.039	0.055	
Breastfeeding < 6 months + Formula	Reference				

*n* = 102 infants; FM%: Fat mass percentage

**Table 4:** Correlates of FMI in infants at 6 months

Variable	B	IC 95%	Eta partial squared	*p*	R^2^ (R^2^ adjusted)
Model 1					0.561 (0.534)
Skinfolds sum	0.211	0.166 – 0.256	0.475	< 0.0001	
Sex [female]	0.626	-0.103 – 1.355	0.030	0.092	
Exclusive/Predominant Breastfeeding	1.093	0.323 – 1.863	0.077	0.006	
Breastfeeding up to 6 months + Formula	0.762	0.082 – 1.442	0.049	0.029	
Breastfeeding < 6 months + Formula	Reference				
Model 2					0.656 (0.631)
Skinfold sum	0.135	0.085 – 0.185	0.235	< 0.0001	
Waist circumference	0.200	0.122 – 0.279	0.216	< 0.0001	
Sex [female]	0.559	-0.091 – 1.208	0.030	0.091	
Exclusive/Predominant Breastfeeding	1.009	0.322 – 1.695	0.083	0.004	
Breastfeeding up to 6 months + Formula	0.521	-0.092 – 1.133	0.029	0.095	
Breastfeeding < 6 months + Formula	Reference				
Model 3					0.483 (0.450)
Triceps Skinfold	0.446	0.330 – 0.562	0.381	< 0.0001	
Sex [female]	0.589	-0.205 – 1.384	0.022	0.144	
Exclusive/Predominant Breastfeeding	1.048	0.210 – 1.885	0.061	0.015	
Breastfeeding up to 6 months + Formula	0.871	0.124 – 1.617	0.053	0.023	
Breastfeeding < 6 months + Formula	Reference				
Model 4					0.635 (0.608)
Triceps Skinfold	0.267	0.153 – 0.380	0.189	< 0.0001	
Waist circumference	0.237	0.162 – 0.312	0.295	< 0.0001	
Sex [female]	0.542	-0.129 – 1.213	0.027	0.112	
Exclusive/Predominant Breastfeeding	0.980	0.273 – 1.687	0.075	0.007	
Breastfeeding up to 6 months + Formula	0.553	-0.085 – 1.191	0.031	0.088	
Breastfeeding < 6 months + Formula	Reference				

*n* = 102 infants, *FMI:* Fat mass index

## Discussion

This is one of the few studies measuring FM at 6 months of age using ADP in a group of healthy term infants. Our results showed that anthropometric measurements are highly correlated with FM in infants. SFS and WC were strongly associated to FM.

In our study, the model that best correlated anthropometry to FM at 6 months of age included SFS + WC. We observed that when more anthropometric variables were included in the model, the strength of the association with FM improved, as other authors have reported [18, 22].

The increase in the prevalence of childhood overweight and obesity worldwide has encouraged the study of body composition during early stages of life [29, 30]. In Mexico, the prevalence of overweight/obesity in children has been increasing over the years, where one out of every three children (5–11 years old) has this problem [31]. Weight is still the most frequently used indicator in clinical practice to evaluate growth and morbidity in this age group. FM measurement should be the basis for obesity diagnosis and to identify metabolic risk. Early detection of alterations in FM appears critical; therefore, FM should be considered an outcome of nutrition and lifestyle interventions in infancy. Predictions equations using different anthropometric measurements for estimating FM in infants are needed and will facilitate this task in clinical settings.

On the other hand, BMI has been proposed to assess growth and nutritional status because it is known to correlate with body fatness and increases the risk of obesity-related diseases in adults [32, 33]. However, some inherent limitations of BMI are that it does not distinguish between FM and FFM, it does not reflect FM distribution and the relative contributions of FM and FFM to body weight vary by ethnicity [12, 34, 35]. So, there are still concerns about its use in pediatric population [35, 36]. In older children (≥7 years old), authors have found some misclassifications of obesity diagnosis using BMI in comparison to body fatness [37, 38]. They stated that the screening ability of BMI varied across race-ethnic groups, and also across the extremes of FM [38]. In infants (1, 4, and 7 months old), Bell et al. [39] found that BMI is limited as a surrogate for adiposity and (especially) adiposity changes. However, there is still controversy [40, 41].

Skinfolds are valid measures of subcutaneous fat and have been used for estimating total body fat in adults, but there is limited information about their validity in childhood and infancy. Bias may be introduced by age, measurement error and error inherent with increasing fatness [10, 32]. In our study, we observed a strong correlation between SFS (BSF, TSF, SSF) and FM (% and index). Other authors have reported that skinfolds are good predictors of FM% in school-age children and, unlike BMI, skinfolds may be useful for both extremes of body fatness [21, 30, 32]. Wohlfahrt-Veje et al. [21] found that FM% estimation from skinfolds (TSF and SSF) showed the highest correlation and best agreement with FM%, measured by dual x-ray absorptiometry, and was superior at identifying children (8–14 years old) with higher adiposity, as compared to only BMI or WC. Tuan et al. [42] reported a higher agreement between FM and TSF and waist-to-height ratio, rather than between FM and BMI. Our models show a slightly better association of SFS with FM, but when availability of measurements is limited, TSF alone could be useful.

WC is a measurement used to assess central adiposity [12, 16]. Considering fat distribution indicators could be important for a more comprehensive FM assessment, as it reflects metabolic risk [43]. Santos et al. [44] found that subcutaneous fat (measured by skinfolds) in infancy (1.5 and 24 months) was positively associated to total and abdominal fat at 6 years old. Some authors have identified moderate-strong associations of WC with total body fat [15, 20, 45], trunk fat in children [22], concluding that WC may be useful to define obesity and to predict relative adiposity in children [15, 46]. This is important because the estimation of FM% and FMI improved when WC was included in the models. The skinfolds that we measured represent subcutaneous fat and two of them (BSF and TSF) are measures of peripheral fat. WC is an indirect measure of visceral fat [12, 16], so including both type of measurements appears to be ideal.

The use of FMI (when compared to FM% alone) has been proposed as a more accurate indicator of adiposity and more sensitive to changes in body fat stores [35, 47], even in infants [48], due to the consideration and normalization to height/length [27, 49]. Infants at potential risk (but with “normal” BMI) could be detected using FMI [48] because it better identifies excess adiposity than FM%, which underestimates it [35]. In our models, stronger associations were found between anthropometry and FMI, in comparison to FM%.

In our study, infants that were exclusive/predominant breastfed during the first 6 months showed higher FM and anthropometric measurements at 6 months. Some authors have reported higher adiposity in exclusively breastfed infants when compared to those that were formula fed at 3–4 and at 6 months of age [6–8].

This study has some limitations. Fat mass measurement at birth was not feasible, therefore, we have no data to correlate in this stage. It is well known that skinfold measurement may introduce a high measurement error; to decrease it, one nutritionist with previous experience and anthropometric training and standardization performed them by duplicate. The small sample and low prevalence of obesity in this group of infants might limit the associations found in this analysis. Performance of anthropometric measures may differ in the extremes of obesity, as has been shown in adults.

## Conclusions

Anthropometric measurements were good determinants of FM at 6 months of age in healthy term infants. Skinfolds sum and waist circumference were the strongest anthropometric variables associated to FM. Type of feeding and sex influence body fat in this age group. Exclusive/predominant breastfeeding was strongly associated with FM.

## Abbreviations

ADP: Air displacement plethysmography
BF + F: Breastfeeding up to 6 months combined with formula feeding
BF < 6 m + F: Breastfeeding less than 6 months combined with formula feeding
BMI: Body mass index
BMI/A: Body-mass-index for age
BSF: Biceps skinfold
E/P BF: Exclusive/Predominant breastfeeding
FFM: Fat-free mass
FM: Fat mass
FM%: Fat mass percentage
FMI: Fat mass index
SFS: Skinfolds sum
SSF: Subscapular skinfold
TSF: Triceps skinfold
W/L: Weight-for-length
WC: Waist circumference
WHO: World Health Organization
WLR: Waist circumference-for-length ratio
